# Laryngismus Stridulus

**Published:** 1890-02

**Authors:** Ella M. Tuttle

**Affiliations:** New Berlin, New York


### LARYNGISMUS STRIDULUS—CLINICAL CASE.

Ella M. Tuttle, M. D., New Berlin, New York.

May 8th, 1889.—Was called to see Minnie A., eight months old. Her mother told me that she had a hard cold in April, and had not been well since, in spite of much allopathic dosing. The doctor had finally told the parents that the child would not probably live to be a year old, so as a last resort they decided to try Homœopathy. On visiting the child I found a well-marked case of *Laryngismus stridulus.* About once in ten minutes the little one would start, throw up her hands, gasp for breath, and become very red (almost purple) in the face. Then after a few moments the air would enter the glottis with the peculiar crowing sound so characteristic of the disease, and the paroxysm would terminate in a short fit of crying. Several times, according to the mother's story, the paroxysms had been so severe that she had picked up the child for dead.

The child was plump and fair looking, had red cheeks and blue eyes. Her appetite was good, bowels regular. She also had the attacks in the night, but much less frequently. From the paroxysmal nature of the disease, the redness of the face, and the crying at the close of the attack I gave a dosè of Bell.$^3$ and left Sac. lac. to be taken every two hours.

May 11th.—The child reported better, the attacks being lighter and not so frequent. Sac. lac.

May 13th.—The attacks seem growing more severe. A dose of Bell.$^3$ followed by Sac. lac.

May 18th.—No better. I now "took the case" again with more care. Learned that for the last month the child had seemed to be losing the use of its lower limbs. On putting my hand on the child's feet I found them *cold* and *clammy*, and on questioning I found that the pillow around the baby's head was usually wet from perspiration in the morning. I had been reading in my Homœopathic Physician the advice to "prescribe for the patient instead of the disease," so, though I could see no pathological relation between Calc-carb. and *laryngismus stridulus*, I gave a dose of Calc-carb. to be followed by Sac. lac., for the amusement of the mother.

May 21st.—The child has had no more paroxysms since the 18th. Sac. lac.

I saw the child occasionally till September, but there was no return of the attacks, and she had begun to bear her weight on her feet. The one dose of Calcarea cured her.

